# 分子印迹聚合物功能化二氧化硅纳米颗粒的合成及其分离识别马兜铃酸

**DOI:** 10.3724/SP.J.1123.2021.06024

**Published:** 2021-10-08

**Authors:** Yuemei ZHANG, Lihua GUO, Yijun LI, Xiwen HE, Langxing CHEN, Yukui ZHANG

**Affiliations:** 1.南开大学化学学院, 生物传感与分子识别天津市重点实验室, 天津 300071; 1. College of Chemistry, Tianjin Key Laboratory of Biosensing and Molecular Recognition, Nankai University, Tianjin 300071, China; 2.中国科学院大连化学物理研究所, 辽宁 大连 116023; 2. Dalian Institute of Chemical Physics, Chinese Academy of Science, Dalian 116023, China

**Keywords:** 表面印迹技术, 二氧化硅纳米颗粒, 马兜铃酸, 中药, surface imprinting technique, silica nanoparticles, aristolochic acids (AAs), traditional Chinese medicine (TCM)

## Abstract

马兜铃酸是马兜铃科植物中含有硝基菲羧酸基团的一类物质,被广泛应用于各种疾病的治疗,研究表明含有马兜铃酸的植物或植物衍生产品对人体有害,需要监测药物中马兜铃酸的存在。分子印迹聚合物对目标物的高亲和力使其特别适合作为吸附剂从混合物中去除和识别目标物。以SiO_2_胶体纳米颗粒为基底,利用表面分子印迹的方法合成了核-壳结构SiO_2_表面印迹纳米颗粒(SiO_2_@MIP NPs)。采用紫外可见光谱研究了模板分子马兜铃酸Ⅰ和功能单体丙烯酸、甲基丙烯酸、2-乙烯基吡啶、丙烯酰胺及甲基丙烯酰胺的作用,发现2-乙烯基吡啶与马兜铃酸Ⅰ的作用最强,被选为制备印迹聚合物的单体。采用傅立叶变换红外光谱仪(FT-IR)、透射电子显微镜(TEM)、热重分析仪、氮气吸附比表面分析仪对分子印迹聚合物进行了表征。TEM显示印迹纳米颗粒的粒径在270 nm左右,分子印迹层的厚度为35 nm,有利于模板分子的传输。TEM、FT-IR和热重分析仪的结果均证明实验成功合成了分子印迹聚合物。实验进一步研究了印迹聚合物SiO_2_@MIP NPs和非印迹聚合物SiO_2_@NIP NPs的吸附性能,并结合SiO_2_@MIP NPs和SiO_2_@NIP NPs的比表面积和孔径测定数据,发现SiO_2_@MIP NPs表面的印迹位点是导致二者吸附差异的主要原因。SiO_2_@MIP NPs和SiO_2_@NIP NPs的动力学吸附表明SiO_2_@MIP NPs具有快的吸附平衡时间(120 s),而且SiO_2_@MIP NPs的吸附行为符合Langmuir单分子层吸附。SiO_2_@MIP NPs的选择性通过印迹因子(IF)和选择性系数(SC)来评价。实验结果表明,SiO_2_@MIP NPs具有高的印迹因子(4.9),对模板结构类似物有较好的选择性,选择系数为2.3~6.6。最后将制备的SiO_2_@MIP NPs作为吸附剂用于加标中药样品川木通的预处理,用HPLC进行分析测定,方法的回收率为73%~83%,实验结果显示SiO_2_@MIP NPs可作为高选择性材料用于中药中马兜铃酸的选择性分离分析。

## Abstract (en)

Aristolochic acids (AAs), which is commonly found in *Aristolochia* and *Asarum* plants, has been widely used in several traditional medicine practices due to their anti-inflammatory, anti-malarial, and anti-hyperglycemic activities. Recently, researchers have found a “decisive link” between liver cancer and aristolochic acid after analyzing a large number of liver cancer samples around the world. Therefore, a highly sensitive and selective method is required for the analysis of AAs in traditional Chinese medicines (TCM). For the determination of AAs in TCM, pretreatment is indispensable because in actual TCM samples, AAs is present in trace amounts and the complex matrix exerts interference. In the past decades, molecularly imprinted polymers (MIPs) have attracted considerable attention as an alternative for the trace analysis in complicated matrices.

In this study, MIP-coated SiO_2_ nanoparticles (SiO_2_@MIP NPs) was prepared for the determination of aristolochic acid by surface molecular imprinting using aristolochic acid Ⅰ (AAI ) as the template molecule, 2-vinylpyridine (VPY) as the functional monomer, and ethyleneglycol dimethacrylate (EGDMA) as the cross-linking agent. Core-shell-structure SiO_2_@MIP NPs were obtained by modifying vinyl groups on the surface of SiO_2_ NPs, coating MIPs films onto the silica surface via selective polymerization, and final extraction of template AAI and generation of the recognition site.

To find a suitable functional monomer for the best imprinting effect, the interaction between the template and the functional monomers, including acrylic acid (AA), methyl acrylic acid (MAA), 2-vinyl pyridine (VPY), acrylamide (AM), and methylacrylamide (MAM) was investigated. Electrostatic interaction between AAI and VPY resulted in the maximum decrease in absorbance of AAI at 250 nm. Therefore, VPY was chosen for the preparation of MIP. The morphological and physical properties of the MIPs were characterized by transmission electron microscopy (TEM), Fourier-transform infrared (FT-IR) spectroscopy, thermogravimetric analysis, and N_2_ adsorption and desorption surface analysis. TEM images showed that SiO_2_ NPs were monodispersed with diameter of about 200 nm. The clear core-shell structure of SiO_2_@MIP NPs was observed, and the thickness of MIPs coating was about 35 nm. The FT-IR spectra of SiO_2_ NPs, vinyl group modified SiO_2_ and SiO_2_@MIP NPs revealed that the vinyl group and organic MIP layer were successfully modified at SiO_2_ sequentially. The results of thermogravimetric analysis were consistent with the FT-IR data for different SiO_2_ NPs. The nitrogen gas adsorption-desorption experiments showed that SiO_2_@MIP NPs and non-imprinted polymer (SiO_2_@NIP NPs) have the same pore volumes, while the surface area and pore size of MIPs were slightly larger than those of NIPs. Therefore, the difference in adsorption between SiO_2_@MIP NPs and SiO_2_@NIP NPs resulted from the imprinted sites on the MIP surface, rather than the difference in their surface areas.

The adsorption properties of SiO_2_@MIP NPs were demonstrated by kinetic, isothermal, and selective adsorption experiments. The results of these experiments displayed that SiO_2_@MIP NPs reached adsorption equilibrium within a short period (120 s) and possessed a much higher rebinding ability than SiO_2_@NIP NPs. To verify the selectivity of SiO_2_@MIP NPs for AAI, three structural analogues (viz. tanshinone ⅡA, 2-methoxy-5-nitrophenol, and benzoic acid) were selected. The results showed that the binding capacity of SiO_2_@MIP NPs was much higher than those of these analogues. SiO_2_@MIP NPs have high adsorption capacity (5.74 mg/g), high imprinting factor (4.9), good selectivity coefficient (2.3-6.6) towards the structural analogues. SiO_2_@MIP NPs was used as an adsorbent and combined with HPLC for the selective separation of AAI in TCM. The recoveries of Kebia trifoliate samples spiked with three levels of AAI (0.3, 0.5, and 1.0 μg/mL) ranged from 73% to 83%. The results suggested that the proposed SiO_2_@MIP NPs could be used for selective enrichment of AAI from real complex TCM samples.

马兜铃酸是衍生自马兜铃科植物、结构含有硝基菲羧酸基团的一类物质。近十几年来,以马兜铃酸为有效成分制成的各种药物被广泛应用于各种疾病的治疗,但是最近有报道^[1,2,3,4]^指出,含有马兜铃酸的植物或植物衍生产品对人具有肾毒性、致突变性和致癌性。因此为了维护人体的健康,需要监测药物中马兜铃酸的存在。目前马兜铃酸的测定方法主要有薄层色谱法、高效液相色谱法、高效液相色谱-质谱法、毛细管电泳法等^[5,6,7,8,9]^。但是实际中药样品中马兜铃酸含量低,且中药成分复杂,增加了马兜铃酸的检测难度。因此开发合理的预处理方法对于从复杂中药样品中提取马兜铃酸十分必要。

分子印迹聚合物(MIPs)是一类对特定物质具有特异性吸附的分离材料。近年来,分子印迹聚合物常作为吸附剂被用于药物、环境污染物、兽药的提取^[10,11,12,13]^。但是大多数MIPs采用传统本体分子印迹方法制备而得,大部分印迹识别位点被包埋在内部,导致其识别位点不均一,传质阻力大,动力学平衡慢。表面分子印迹技术由于印迹效率高、传质速率快成为分子印迹中应用广泛的技术。在表面分子印迹的基底中,SiO_2_胶体纳米颗粒(SiO_2_ NPs)表面富含羟基易于官能化反应,而且合成SiO_2_ NPs的条件简单、方法已经十分成熟,另外SiO_2_ NPs具有化学性质稳定、机械强度较高、耐热、耐腐蚀、无毒性且生物相容性较好等优点,使其成为最常用的核-壳型分子印迹聚合物的核^[14,15,16]^。目前以马兜铃酸为模板的分子印迹聚合物的研究^[9,17,18]^报道不多。本课题组前期^[9]^采用简便、绿色的溶胶-凝胶方法合成磁性碳纳米管MIPs,并用于中药中马兜铃酸Ⅰ(AAI)的检测分析。Xiao等^[17]^采用可逆加成-断裂链转移聚合(RAFT)制备MIPs并用于AAI的去除。Ge等^[18]^合成一种磁性介孔碳分子印迹聚合物,并作为磁性固相萃取吸附剂选择性识别大鼠尿液中的马兜铃酸Ⅰ和Ⅱ。

本工作以SiO_2_ NPs为核,以AAI为模板分子,2-乙烯基吡啶(VPY)为功能单体,乙二醇二甲基丙烯酸酯(EGDMA)为交联剂,利用表面分子印迹的方法合成分子印迹聚合物包覆的SiO_2_纳米颗粒(SiO_2_@MIP NPs)。将此SiO_2_@MIP NPs作为吸附剂,并与HPLC相结合,用于中药中AAI的选择性分离和识别。

## 1 实验部分

### 1.1 仪器与试剂

SPD-M20A高效液相色谱仪(配置两台LC-20AD泵、一台在线脱气机DGU-20A5以及一个二极管阵列检测器,色谱数据的采集和处理在LCsolution工作站完成)、UV-2450型紫外/可见分光光度计(日本岛津公司); G2 F20透射电子显微镜(美国FEI公司); AVATAR 360傅里叶红外光谱(美国Nicolet公司); NETZSCH-TG209C热重分析仪(德国NETZSCN公司); ASAP 2460型N_2_吸附表面分析仪(美国麦克公司);实验用高纯水由AWL-0502-U艾科浦超纯水系统(重庆颐洋企业发展有限公司)提供。

AAI购自天津希恩思奥普德科技有限公司;丙烯酸(AA)购自上海阿拉丁生化科技股份有限公司;甲基丙烯酸(MAA)购自天津艾利安电子科技有限公司;丙烯酰胺(AM)、甲基丙烯酰胺(MAM)、四乙氧基硅烷(TEOS)、甲基丙烯酰氧丙基三甲氧基硅烷(MPS)、EGDMA及偶氮二异丁腈(AIBN)购自阿法埃莎(中国)化学有限公司;2-甲氧基-5-硝基苯酚(MENP)购自北京华威锐科化工有限公司;丹参酮ⅡA(TAN ⅡA)和苯甲酸(BA)购自上海麦克林生化科技有限公司;VPY购自凯玛特(天津)化工科技有限公司;中药川木通购自安国新药都药材市场。其他化学试剂均为分析纯及以上级别。

### 1.2 核-壳结构马兜铃酸分子印迹聚合物纳米颗粒的制备

1.2.1 双键修饰的SiO_2_ NPs(SiO_2_@MPS NPs)的制备

利用传统的Stöber法首先制备SiO_2_ NPs^[19]^。具体步骤如下:量取170 mL无水乙醇至250 mL圆底烧瓶中,快速加入9.40 mL TEOS,磁力搅拌,转速为600 r/min;油浴加热至40 ℃,搅拌30 min后,用恒压滴液漏斗将4.5 mL蒸馏水和12 mL氨水的混合液缓慢滴入烧瓶中,保持温度反应12 h,得到乳白色SiO_2_胶体纳米颗粒分散液;将SiO_2_胶体纳米颗粒分散液以8000 r/min转速离心,弃去上清液,离心产物再次分散到无水乙醇中,反复离心、分散循环;最后一次离心得到SiO_2_ NPs。

称取400 mg SiO_2_ NPs,加入到含有20 mL超纯水和80 mL无水乙醇的三口烧瓶中,超声30 min使SiO_2_ NPs分散均匀;开启机械搅拌,以500 r/min的转速剧烈搅拌;依次加入3 mL氨水和1.6 mL MPS,置于65 ℃油浴中反应24 h;所得白色产物以800 r/min的转速离心,弃去上清液,离心产物反复用无水乙醇分散、离心循环3次,最后一次离心产物于40 ℃真空干燥6 h,得到SiO_2_@MPS NPs。

1.2.2 SiO_2_@MIP NPs的制备

称取13.6 mg(0.04 mmol)模板分子AAI,置于50 mL离心管中,加入40 mL甲醇,超声溶解得到深黄色透明液体;在该溶液中加入0.16 mmol VPY,超声进行预聚合;然后将100 mg SiO_2_@MPS NPs完全分散在上述溶液中;再将150 μL(0.8 mmol)EGDMA和20 mg AIBN转移至离心管中,混合,将离心管中的液体全部转移到100 mL三口烧瓶中,冰浴中通入N_2_除氧,持续30 min;除氧后将三口烧瓶密封好,磁力搅拌800 r/min,油浴加热,于50 ℃预聚合1 h,于60 ℃反应24 h,得到的产物为乳黄色悬浊液。将产物离心分离,弃去上清液,离心产物用甲醇重新分散并再次离心分离,上述过程重复直至上清液在320 nm下无紫外吸收,证明已经除去模板分子AAI;将最终所得产物于40 ℃真空干燥6 h,最终得到SiO_2_@MIP NPs。

作为对照,用同样的方法合成非印迹聚合物SiO_2_@NIP NPs,只是不加入模板分子AAI。

### 1.3 SiO_2_@MIP NPs选择吸附性能的研究

将5 mg SiO_2_@MIP NPs分别置于1.5 mL含不同浓度AAI的乙腈溶液中,超声2 min使NPs完全分散,振荡10 min;随后以10000 r/min的转速高速离心,于320 nm处用紫外可见分光光度计测定上清液中AAI浓度,计算SiO_2_@MIP NPs的吸附容量。

选择TAN ⅡA、BA、MENP作为马兜铃酸的结构类似物进行SiO_2_@MIP NPs选择性吸附的探究。分别配制1.5 mL含25.0 μg/mLTAN ⅡA、BA、MENP和AAI的乙腈溶液。称取5 mg SiO_2_@MIP NPs置于上述4种溶液中,超声2 min充分分散后,于室温振荡10 min,高速离心后取上清液用紫外可见分光光度计测定吸光度并计算吸附容量。

### 1.4 SiO_2_@MIP NPs在中药实际样品中的应用

参照Li等^[9]^的方法,称取川木通粉末1 g,置于250 mL单口烧瓶中,加入80 mL甲醇和20 mL超纯水,于80 ℃下回流3 h,收集回流液,重新加入80 mL甲醇、20 mL超纯水后,再次于80 ℃回流3 h。重复4个循环,将所有回流液经过旋蒸后得到棕黄色固体。棕黄色固体超声分散在20 mL甲醇(色谱纯)中,离心除去不能溶解的固体,提取液经0.1 μm有机微孔滤膜过滤后进行HPLC检测。

### 1.5 分析条件

色谱柱为Shim-pack VP-ODS C18柱(150 mm×4.6 mm, 5 μm),柱温为25 ℃,流动相为甲醇-0.1%乙酸水溶液(3;1, v/v),等度洗脱,流速为0.5 mL/min,检测波长为320 nm,进样量为10 μL。

## 2 结果与讨论

### 2.1 SiO_2_@MIP NPs制备条件的优化

SiO_2_@MIP NPs的制备过程如图1所示。先以Stöber法^[19]^合成SiO_2_ NPs,然后以含有双键的硅烷试剂MPS为反应物,经过溶胶-凝胶反应在SiO_2_ NPs表面形成双键基团,即可与功能单体作用,SiO_2_ NPs作为载体,具有较小的粒径和较高的比表面积,更易于对模板分子的吸附。待模板分子洗脱后,得到表面含有印迹位点的SiO_2_@MIP NPs。

在进行分子印迹的过程中,模板分子和功能单体之间作用力越强,越有利于二者形成预聚合混合物后的印迹过程。因此选择合适的功能单体,对提高印迹效果十分重要。AAI的分子结构中具有羧基,因而呈现一定的酸性。AA和MAA属于酸性的功能单体,与同样呈现酸性的AAI之间的结合力较弱;AM和MAM的结构中有氨基,而氨基具有吸电子能力,因此可以与AAI中的羧基通过氢键、静电作用形成可逆的复合物;VPY作为碱性功能单体中的一种,其吡啶环上的氮原子带有正电荷离子,可与羧基中的氢离子发生静电作用,所以理论上VPY更适合作为印迹AAI的功能单体。

**图1:**
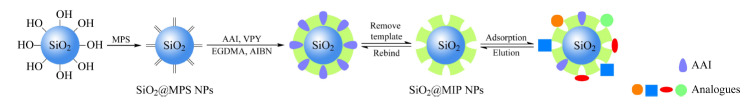
SiO_2_@MIP NPs合成示意图

从功能单体AA、MAA、AM、MAM、VPY分别与AAI混合的预聚合溶液的紫外可见光谱图发现,在功能单体与模板分子比例相同时,VPY与AAI的混合液在250 nm的吸光度较AAI溶液的吸光度下降的最多(见图2a),说明VPY更容易与AAI形成稳定的复合物。为了进一步验证功能单体与AAI的作用,选取VPY按照不同的物质的量之比与AAI混合,当增加VPY的物质的量时,功能单体与模板分子的混合溶液的吸光度随之下降(见图2b),而AAI和AA的混合溶液的吸光度随AA物质的量的增加并没有发生明显的变化(见图2c),实验结果进一步证明了VPY与AAI具有强的非共价作用力。因此,在后续实验中选择VPY作为功能单体。

**图2:**
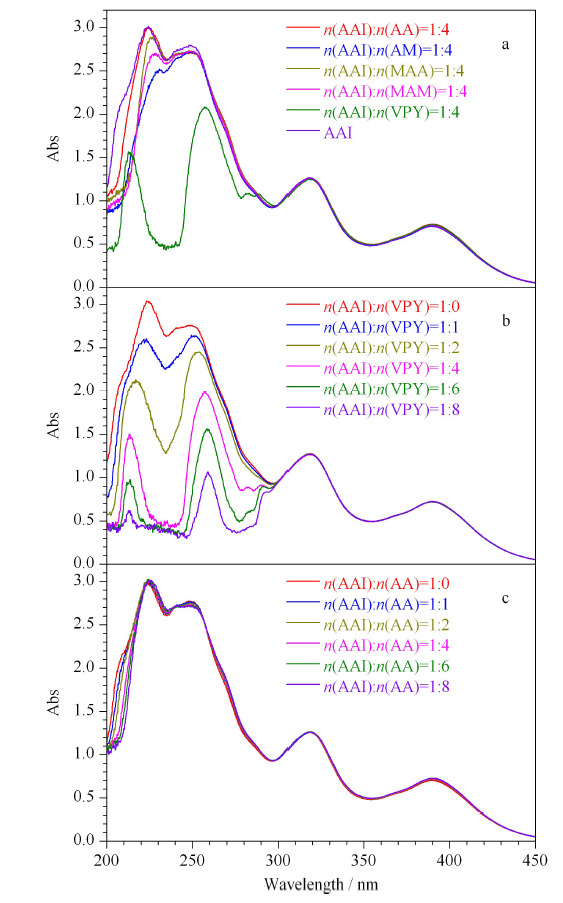
马兜铃酸和不同功能单体混合溶液的紫外可见光谱图

### 2.2 SiO_2_@MIP NPs的性能表征

采用透射电子显微镜对合成的SiO_2_ NPs和SiO_2_@MIP NPs进行形貌表征。如图3所示,SiO_2_ NPs和SiO_2_@MIP NPs呈规整的球形结构,表明表面印迹后球形形貌得到了保留,较大的比表面积有利于后续较快的传质过程。SiO_2_ NPs粒径约为200 nm,微球大小均一,具有很好的单分散性(见图3a)。制备的SiO_2_@MIP NPs呈明显的核-壳结构,SiO_2_胶体纳米颗粒核完全被印迹聚合物壳层包裹(见图3b),表明在SiO_2_胶体微球表面成功包覆上了印迹聚合物,得到的印迹层包裹均匀,厚度约为35 nm。

**图3:**
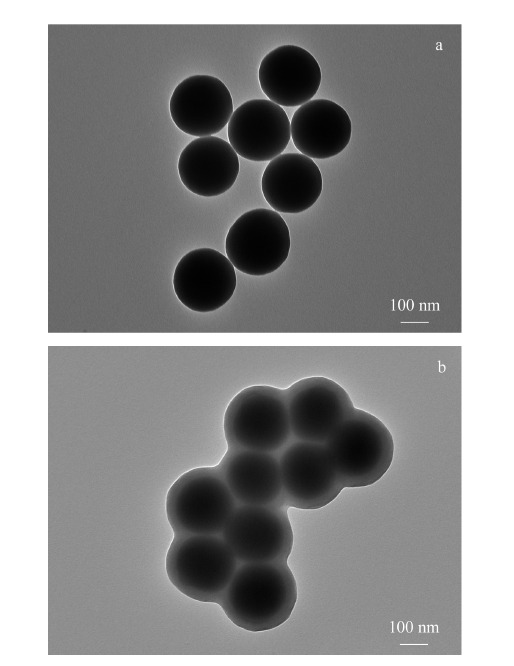
(a)SiO_2_ NPs和(b)SiO_2_@MIP NPs的透射电镜图

通过傅立叶变换红外光谱仪的测定,得到了SiO_2_ NPs、SiO_2_@MPS NPs和SiO_2_@MIP NPs表面官能团分布的信息。图4a中3条FT-IR谱图中均在1089 cm^-1^处出现了吸收峰,对应Si-O-Si的不对称伸缩振动峰;3430 cm^-1^处出现的宽峰是由Si-OH的伸缩振动引起。在SiO_2_@MPS NPs的谱图中,在1623 cm^-1^和1708 cm^-1^处出现了新特征峰,分别对应MPS中C=C振动峰和C=O振动峰,说明已经成功在SiO_2_胶体微球表面修饰双键。在SiO_2_@MIP NPs谱图中,1461 cm^-1^处的吸收峰与EGDMA中与醚键相连的亚甲基中C-H的特征振动有关;2935 cm^-1^处的吸收峰对应着饱和C-H伸缩振动峰;与SiO_2_@MPS NPs谱图相比,由于EGDMA中C=O的引入,使得SiO_2_@MIP NPs谱图中C=O的吸收峰强度大大增加;在SiO_2_@MIP NPs谱图中C=C峰强度也有所降低,是由于SiO_2_@MPS NPs表面上的C=C与功能单体VPY、交联剂EGDMA进行了共聚反应。

**图4:**
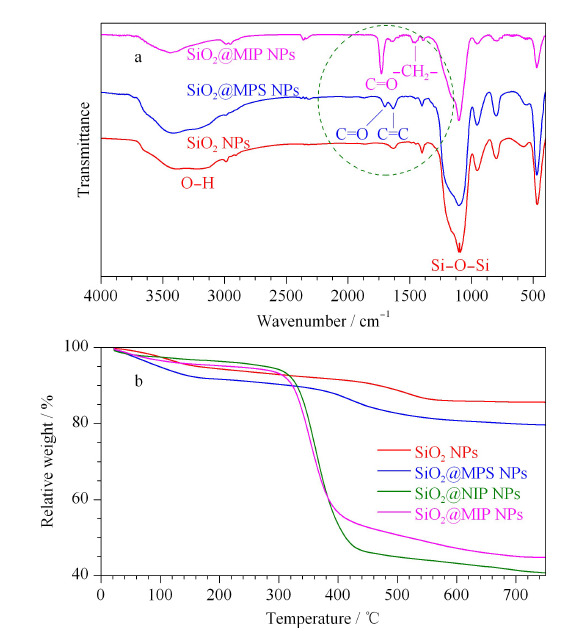
SiO_2_ NPs、SiO_2_@MPS NPs和SiO_2_@MIP NPs的(a)傅里叶红外谱图和(b)热重分析谱图

利用热重分析仪分析了SiO_2_ NPs、SiO_2_@MPS NPs、SiO_2_@MIP NPs和SiO_2_@NIP NPs的热稳定性,结果进一步证实SiO_2_ NPs表面成功包裹了聚合物。图4b的热重分析结果显示SiO_2_ NPs失重约为15%,归因于SiO_2_ NPs所含水分的蒸发和表面羟基的分解;SiO_2_@MPS NPs约有19%的失重,比SiO_2_ NPs的失重增加了约4%,归因于硅球表面成功接枝的双键;SiO_2_@MIP NPs从300 ℃开始发生明显的失重,失重约56%,比SiO_2_@MPS NPs增加了约37%,这归因于表面分子印迹层的分解,进一步证明了硅球表面聚合物层的存在;SiO_2_@NIP NPs从300 ℃开始也存在明显的失重,失重约为60%,略高于SiO_2_@MIP NPs。在500 ℃之后,SiO_2_ NPs、SiO_2_@MPS NPs、SiO_2_@MIP NPs和SiO_2_@NIP NPs没有继续发生明显的失重,说明材料均具备热稳定性。

对SiO_2_@MIP NPs和SiO_2_@NIP NPs进行了比表面积和孔径的分析。SiO_2_@MIP NPs和SiO_2_@NIP NPs的N_2_吸附-脱附曲线非常类似(见图5),说明二者的物理吸附没有太大差别。采用Brunauer-Emmett-Teller (BET)方程和Barrett-Joyner-Halenda (BJH)方法分析了SiO_2_@MIP NPs和SiO_2_@NIP NPs的比表面积和孔径分布参数,SiO_2_@MIP NPs的比表面积(15.56 m^2^/g)稍微高于SiO_2_@NIP NPs的比表面积(14.49 m^2^/g),二者的孔径大小相同(1.590 nm), SiO_2_@MIP NPs的孔容(0.01820 cm^3^/g)略大于SiO_2_@NIP NPs(0.01765 cm^3^/g)。结合热重分析结果,说明SiO_2_@NIP NPs表面包覆上了更多的聚合物。因此,可以认为MIPs表面的印迹位点是导致吸附差异的主要原因,而不是由于制备材料比表面积的不同。

**图5:**
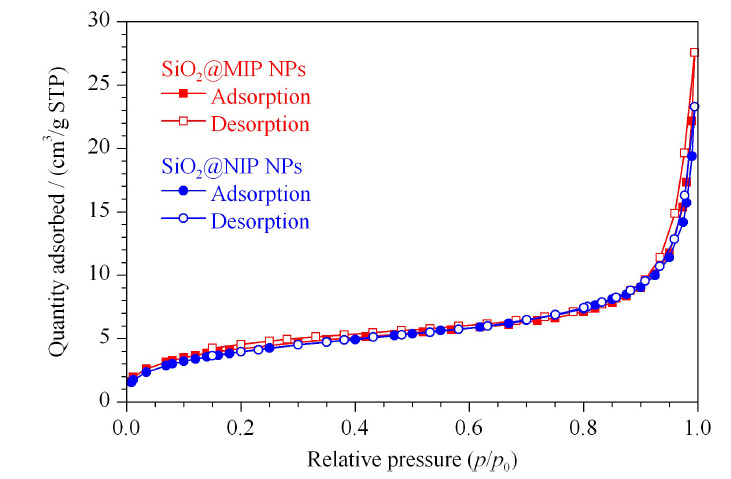
SiO_2_@MIP NPs和SiO_2_@NIP NPs的N_2_吸附-脱附等温曲线

### 2.3 SiO_2_@MIP NPs的吸附性能

SiO_2_@MIP NPs和SiO_2_@NIP NPs对马兜铃酸的动力学吸附如图6a所示。从图中可以看出,吸附剂的吸附量随着时间的增加逐渐增大,前60 s吸附量增加明显,归因于吸附剂表面的印迹位点尚未被完全占据;在大约120 s时吸附量基本保持不变,证明达到了吸附平衡。快速的吸附平衡证明了采用表面印迹方法合成的SiO_2_@MIP NPs具有快速的传质过程和结合能力。此外,还观察到SiO_2_@MIP NPs的吸附量明显大于SiO_2_@NIP NPs,说明相比于SiO_2_@NIP NPs的非特异性吸附,SiO_2_@MIP NPs的特异性吸附发挥了作用。

**图6:**
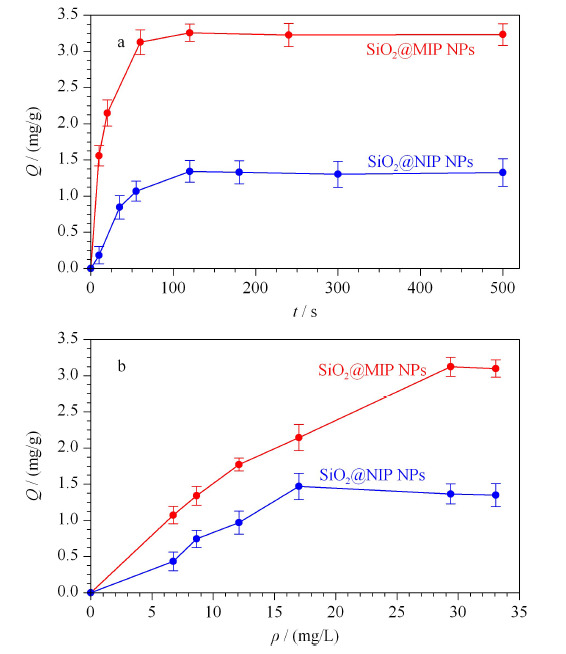
SiO_2_@MIP NPs和SiO_2_@NIP NPs的(a)动力学吸附曲线和(b)热力学吸附曲线(*n*=3)

为了进一步研究所制备印迹材料的吸附性能,SiO_2_@MIP NPs和SiO_2_@NIP NPs对不同初始浓度AAI标准溶液进行了等温吸附平衡实验,从图6b中可以观察到,SiO_2_@MIP NPs出现吸附平衡时对应的AAI浓度较高,且SiO_2_@MIP NPs的吸附量明显大于SiO_2_@NIP NPs,这也印证了SiO_2_@MIP NPs具有与AAI相匹配的特异性识别位点,能够结合更多的AAI。对SiO_2_@MIP NPs的热力学吸附曲线分别做了Langmuir拟合和Freundlich拟合,二者均具有较高的拟合程度,可能是因为SiO_2_@MIP NPs除了依靠氢键结合AAI外,还有离子间相互作用。由于Langmuir拟合(*r*=0.9966)优于Freundlich拟合(*r*=0.9933),因此推断吸附剂的吸附行为为单分子层吸附,主要发生在吸附剂的表面且吸附剂具有均匀的与AAI相匹配的结合位点。根据Langmuir拟合方程,得出理论最大吸附量为5.74 mg/g。

在实际中药样品分析中,复杂的基质干扰往往会影响分子印迹聚合物的识别结果。因此,在识别过程中需要分子印迹聚合物具有较好的选择性。根据AAI的分子结构,实验分别选择了具有多苯环结构的丹参酮ⅡA,具有硝基的2-甲氧基-5-硝基苯酚和具有酸性的苯甲酸作为AAI的结构类似物。SiO_2_@MIP NPs的吸附选择性通过目标物在印迹聚合物颗粒和溶液之间的分配系数(*K*)、印迹因子(IF)和选择性系数(SC)来评价^[20]^。实验结果表明,SiO_2_@MIP NPs具有高的印迹因子(4.9),对模板结构类似物有较好的选择性,选择系数为2.3~6.6。

对比SiO_2_@MIP NPs或SiO_2_@NIP NPs对AAI及其结构类似物吸附的结果(见图7),发现SiO_2_@MIP NPs对模板分子AAI的吸附量显著高于其他3种类似物,此外对AAI的IF也比其他类似物更高,这表明SiO_2_@MIP NPs表面的印迹层具有特定的识别位点,这些位点在大小、形状和功能基团方面都能够和AAI匹配互补,因而模板AAI能够比其他3种类似物更容易占据所形成的空腔。在3种结构类似物中,无论SiO_2_@NIP NPs还是SiO_2_@MIP NPs,对苯甲酸的吸附量均较高,这可能是因为苯甲酸中的羧基和功能单体VPY有类似于AAI和VPY之间的离子间作用力,因此导致了较高的非特异性吸附。对比SiO_2_@NIP NPs对AAI、TAN ⅡA、BA和MENP的吸附量,可以发现SiO_2_@NIP NPs对AAI的吸附量最高。这一实验结果与Baggiani等^[21]^的报道一致,即如果NIP与目标分子也有结合特性,则同样方法合成的MIP表现出的印迹效应将更为显著。

**图7:**
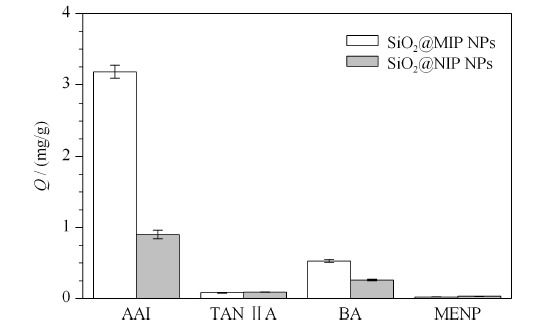
SiO_2_@MIP NPs和SiO_2_@NIP NPs对AAI、TANⅡA、BA和MENP的吸附容量(*n*=3)

样品预处理吸附剂的一个显著特点是具有良好的可重复使用性。因此,研究制备的分子印迹材料的再生能力具有重要意义。以1%乙酸甲醇溶液为洗脱液,进行了5次吸附-再生循环吸附试验,发现SiO_2_@MIP NPs对AAI在5次吸附循环后,其吸附量与第一次相比损失了约16%。这是因为随着洗脱次数的增加,印迹聚合物表面的特异性结合位点有可能被破坏。经过5次吸附-再生循环吸附试验,印迹聚合物的吸附容量虽有降低,但是仍然超过第1次吸附容量的80%,可以认为所制备的印迹聚合物材料具有良好的重复使用性能,可作为吸附剂用于实际样品中AAI的富集和检测分析。

### 2.4 SiO_2_@MIP NPs对AAI的选择性吸附

将制备的SiO_2_@MIP NPs用作吸附剂并结合HPLC对加标中药川木通样品进行分析。在0.050~200.0 μg/mL范围内,AAI浓度与其色谱峰面积具有较好的线性关系(*r*=0.9999),该方法的检出限(LOD)和定量限(LOQ)分别为0.033 μg/mL和0.11 μg/mL。加入AAI到川木通提取液中,使AAI的加标水平分别为0.3、0.5和1.0 μg/mL,通过HPLC检测,发现AAI的加标回收率为73%~83%,相对标准偏差(RSD)小于3.5%。

将SiO_2_@MIP NPs加入到中药川木通的提取液中,分析后,相应的色谱图如图8所示。没有加AAI的川木通样品在保留时间为17.5 min处没有出现色谱峰;而加标1 μg/mL的AAI川木通样品中出现了AAI的色谱峰;用SiO_2_@MIP NPs吸附剂对加标的川木通样品进行吸附后,发现AAI的色谱峰消失,这证明合成的SiO_2_@MIP NPs具有对AAI的特异性吸附,并能够用于中药样品中AAI的选择性去除。

**图8:**
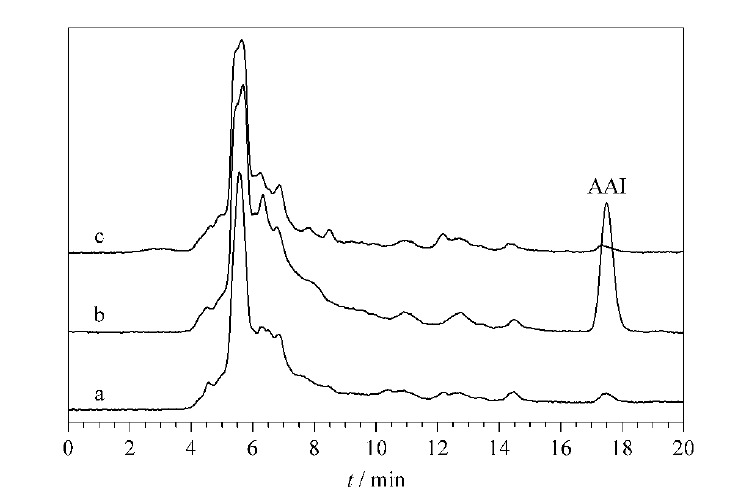
川木通提取液的色谱图

## 3 结论

本工作采用表面分子印迹技术制备了核-壳结构的马兜铃酸分子印迹聚合物,并作为吸附剂应用于中药川木通中AAI的选择性去除。一系列吸附实验表明,制备的分子印迹聚合物对AAI的吸附平衡时间短、吸附容量理想、选择性高。本文建立了SiO_2_@MIP NPs作为吸附剂的HPLC方法,并应用于中药中马兜铃酸的选择性分离分析。基于核-壳结构SiO_2_@MIP NPs,可作为高选择性材料用于中药有效成分的分离富集和化学危害物质的去除。
